# Chemosensitization of cancer cells by siRNA using targeted nanogel delivery

**DOI:** 10.1186/1471-2407-10-10

**Published:** 2010-01-11

**Authors:** Erin B Dickerson, William H Blackburn, Michael H Smith, Laura B Kapa, L Andrew Lyon, John F McDonald

**Affiliations:** 1 School of Biology, Georgia Institute of Technology, 310 Ferst Drive, Atlanta, GA, 30332, USA; 2 Petit Institute for Bioengineering and Bioscience, Georgia Institute of Technology, 315 Ferst Drive, Atlanta, GA, 30332, USA; 3 Ovarian Cancer Institute, Georgia Institute of Technology, 315 Ferst Drive, Atlanta, GA, 30332, USA; 4 Department of Chemistry and Biochemistry, Georgia Institute of Technology, 901 Atlantic Drive, Atlanta, GA, 30332, USA; 5 Veterinary Clinical Sciences Department, University of Minnesota, 1352 Boyd Avenue, St. Paul, MN, 55108, USA; 6 Department of Cellular and Molecular Medicine, Johns Hopkins School of Medicine, 1830 E. Monument Street, Baltimore, MD, 21205, USA

## Abstract

**Background:**

Chemoresistance is a major obstacle in cancer treatment. Targeted therapies that enhance cancer cell sensitivity to chemotherapeutic agents have the potential to increase drug efficacy while reducing toxic effects on untargeted cells. Targeted cancer therapy by RNA interference (RNAi) is a relatively new approach that can be used to reversibly silence genes *in vivo *by selectively targeting genes such as the epidermal growth factor receptor (EGFR), which has been shown to increase the sensitivity of cancer cells to taxane chemotherapy. However, delivery represents the main hurdle for the broad development of RNAi therapeutics.

**Methods:**

We report here the use of core/shell hydrogel nanoparticles (nanogels) functionalized with peptides that specially target the EphA2 receptor to deliver small interfering RNAs (siRNAs) targeting EGFR. Expression of EGFR was determined by immunoblotting, and the effect of decreased EGFR expression on chemosensitization of ovarian cancer cells after siRNA delivery was investigated.

**Results:**

Treatment of EphA2 positive Hey cells with siRNA-loaded, peptide-targeted nanogels decreased EGFR expression levels and significantly increased the sensitivity of this cell line to docetaxel (P < 0.05). Nanogel treatment of SK-OV-3 cells, which are negative for EphA2 expression, failed to reduce EGFR levels and did not increase docetaxel sensitivity (P > 0.05).

**Conclusion:**

This study suggests that targeted delivery of siRNAs by nanogels may be a promising strategy to increase the efficacy of chemotherapy drugs for the treatment of ovarian cancer. In addition, EphA2 is a viable target for therapeutic delivery, and the siRNAs are effectively protected by the nanogel carrier, overcoming the poor stability and uptake that has hindered clinical advancement of therapeutic siRNAs.

## Background

Although a number of chemotherapeutic treatments have been shown to be effective at inhibiting or eliminating cancer cell growth in preclinical studies, clinical applications are often limited due to the toxic side effects associated with anticancer drugs. Patients are often unable to tolerate the level of a drug needed to effectively eliminate malignant cells while levels that can be tolerated are insufficient therapeutically. As a result, chemoresistance and subsequent tumor recurrence are often the outcome of such therapies. An example of this all too common event is the use of taxanes (paclitaxel and its semi-synthetic analogue, docetaxel) in the treatment of a variety of cancers including ovarian, breast, prostate, and non-small cell lung cancers [1,2]. While surgery along with taxane- and platinum-based chemotherapy for advanced ovarian cancer has allowed up to 80% of women to achieve a clinical response [3], cancers in most patients initially diagnosed with late stage disease eventually recur.

Development of methods to circumvent resistance may ultimately improve the impact of adjuvant therapy, resulting in prolonged disease-free intervals and survival. Novel targeted therapies that interfere with specific molecular signaling pathways affecting cancer cell survival are being developed as potential treatment options to render cancer cells more sensitive to cytotoxic chemotherapy. Targeted therapies that increase cancer cell sensitivity to chemotherapies offer the benefits of lowering unwanted side effects and increasing the likelihood of destroying resistant cells while avoiding healthy cells where there is little or no expression of the targeted entity.

Recent studies have shown that sensitivity of ovarian cancer cells to the taxane, paclitaxel, is enhanced when the drug is administered in combination with an inhibitor of EGFR. EGFR and its ligand, epidermal growth factor (EGF), play critical roles in the progression of ovarian cancer through their effects on cellular proliferation, apoptosis, angiogenesis, and metastasis [4-6]. EGFR is overexpressed or dysregulated in many solid tumors [7-10], and high levels are expressed in 33-98% of all epithelial ovarian cancers [11-14]. Their high expression is believed to mitigate the effectiveness of taxane chemotherapy by inhibiting cell division and apoptosis [15-17]. Reports of inhibition of EGFR with tyrosine kinase inhibitors (TKI) [e.g. gefitinib (Iressa)] and monoclonal antibodies (e.g. cetuximab) have demonstrated that silencing of receptor activity increases chemosensitization of tumor cells including ovarian cancer cells [6,18-22]. While targeting EGFR as well as other members of the human EGFR (HER) family has proven successful, not all tumors that are expected to respond to these agents do so. Often, emergence of drug resistance occurs either by targeted mutation [23,24] or induction of alternative signaling pathways [24,25]. These results highlight the need for further targeted approaches.

Based on these findings, we sought to determine if siRNA against EGFR could be selectively delivered to ovarian cancer cells using a nanoparticle carrier. Targeted cancer therapy by RNA interference (RNAi) is a relatively new approach, and silencing EGFR by RNAi has already shown promising results [26-30]. We report here application of a novel and highly efficient method for the targeted delivery of EGFR siRNA to ovarian cancer cells. The method is based on core/shell hydrogel nanoparticle (nanogel) siRNA carriers, which represent a convenient and versatile structure for targeted drug delivery. The reader is referred to work from our groups for more detailed information regarding the nanocarrier [26,31,32]. These core/shell nanogels are composed mainly of poly(alkylacrylamides), which can be easily synthesized via multi-stage, free-radical initiated precipitation polymerization [31]. In this fashion, a porous hydrogel core appropriate for the entrapment of macromolecular therapeutics can be coated with a porous hydrogel shell that displays the appropriate chemoligation sites for the attachment of targeting ligands. We used a previously described 12 amino acid peptide (**YSA**YPDSVPMMS or YSA) [33] coupled to the surface of ~100-nm diameter core/shell nanogels [composed of poly(*N*-isopropylmethacrylamide) (pNIPMAm) cross-linked with *N, N'*-methylene(bisacrylamide)] [26,34] to permit cell-specific targeting, and the subsequent delivery of high concentrations of EGFR siRNA. The YSA peptide mimics the ligand ephrin-A1, which binds to the erythropoietin-producing hepatocellular (Eph) A2 receptor, while the core/shell nanogel offers an efficient vehicle for cell entry, a protective environment for the siRNA, and a depot for its controlled release. Delivery of nanogel-loaded EGFR siRNA to EphA2 positive cells resulted in the loss of EGFR expression followed by a significant increase in the sensitivity of the targeted cells to docetaxel. Our results indicate that this approach may lead to considerable improvements in the treatment of ovarian and other cancers by increasing the efficacy of chemotherapy while simultaneously reducing the associated negative side effects.

## Methods

All materials were purchased from Sigma-Aldrich (St Louis, MO) and used as received unless otherwise noted.

### Nanogel synthesis

For the present studies, we utilized a nanogel structure that we have previously shown to have excellent siRNA encapsulation and release properties in the context of *in vitro *delivery [26]. The synthesis of the nanogels has been described previously [26,35]. Briefly, nanogel core particles were synthesized by free-radical precipitation polymerization using a molar composition of 98% *N*-isopropylmethacrylamide (NIPMAm), 2% *N, N'*-methylenebis(acrylamide) (BIS) and a small amount (~0.1 mM) acrylamidofluorescein (AFA) to render the nanogels fluorescent for visualization. The core nanogels were then used as seeds for the addition of a hydrogel shell [31,35]. The shell composition was 97.5% NIPMAm, 2% BIS, and 0.5% aminopropylmethacrylamide (APMA, Polysciences, Warrington, PA). The APMA co-monomer was included to provide chemoligation sites for peptide immobilization.

### Peptide conjugation

The YSA peptide (GenScript Corporation, Piscataway, NJ) was conjugated to the nanogels via maleimide coupling to the cysteine residue on the C-terminal end of the peptides, as described [26]. Maleimide-functionalized nanogels were prepared via EDC coupling of ε-maleimidocaproic acid (EMCA) to the primary amines in the nanogel shell. Peptide coupling was performed by introducing the YSA peptide in a 1:1 molar ratio with amine (YSA molecular weight = 1450.66 g/mol). The YSA peptide was then conjugated to the nanogels via maleimide coupling to the cysteine residue on the C-terminal end of the peptides.

### Cell culture

Hey cells were provided by Gordon W. Mills, Department of Systems Biology, the University of Texas, M. D. Anderson Cancer Center. Hey cells were cultured in RPMI 1640 (Mediatech, Manassas, VA) supplemented with 10% v/v heat-inactivated fetal calf serum (Invitrogen, Carlsbad, CA), 2 mM L-glutamine (Mediatech), 10 mM HEPES buffer (Mediatech), penicillin (100 U/ml), and streptomycin (100 μg/mL). SK-OV-3 cells were from the National Cancer Institute and were propagated in McCoy's 5A with L-glutamine (Mediatech) supplemented with 10% v/v heat-inactivated fetal calf serum (Atlanta Biologicals, Lawrenceville, GA), penicillin, and streptomycin (Mediatech).

### RNA encapsulation

Hydrogels were loaded with siRNA as previously described [26]. Briefly, lyophilized nanogels were reswollen in the presence of the siRNA, thereby imbibing the solute within the hydrogel network. In a typical procedure, a 20 μM solution (250 μL) of EGFR siRNA (Dharmacon, Lafayette, CO) was prepared in phosphate buffered saline (PBS). Nanogels were resuspended in this mixture at a concentration of 4 mg per 250 μL of siRNA solution and allowed to shake overnight at room temperature. After the siRNA was encapsulated in the nanogels, they were centrifuged and resuspended to a final concentration of 10 mg/mL in cell culture medium or PBS. Based on this procedure, the final concentration of siRNA was determined to be 16.6 μg siRNA/mg of nanogels. For experiments using a non-specific siRNA, siGLO (Dharmacon) was incorporated into nanogels at the same concentration described for EGFR siRNA.

### Immunoblotting

Hey or SK-OV-3 cells were plated into 6-well cell culture plates (5 × 10^5 ^cells/well), and the cells allowed to adhere overnight at 37°C in a 5% CO_2 _atmosphere. After washing the wells with PBS and replacing the medium, EGFR siRNA-loaded/YSA-conjugated nanogels were added to the wells. Cells were incubated for four hours, washed with PBS, and fresh medium was added to the cells. The cells were incubated at 37°C and 5% CO_2 _in wells for 24, 48, 72, 96, and 120 hours. Control wells were set up to include non-targeted/siRNA-encapsulated pNIPMAm particles, unloaded pNIPMAm particles, YSA alone, and untreated cells. Cells were lysed after the designated time points, and immunoblotting was carried out as described [26]. To determine the optimal concentration of EGFR-siRNA needed for efficient reduction of EGFR expression, the nanogel loading procedure described above was used, but the concentration of particles delivered to each well was altered. The initial concentration of siRNA-encapsulated particles (1 mg/mL of nanogels/5 × 10^5 ^cells) used for the time point experiments was added to the first well. The concentration of subsequent wells was reduced by 10 fold each, resulting in nanogel concentrations of 100, 10, and 1 μg/mL per 5 × 10^5 ^cells. After four hours of incubation with the nanogels, the cells were washed with PBS, and the medium was replaced. The cells were then incubated for an additional 48 hours, and the samples prepared for immunoblotting as described [26].

### Treatment with docetaxel

Hey or SK-OV-3 cells were plated in 96-well cell culture plates at a concentration of 1 × 10^4 ^cells/well. Hey or SK-OV-3 cells were subjected to nanogel delivery of siRNA at nanogel concentrations of 1000, 100, 10, and 1 μg/mL. Forty-eight hours after siRNA delivery, docetaxel was added to Hey or SK-OV-3 cells at concentrations ranging from 0.01-1000 nM. Treatment wells were set up in triplicate, and the cells were incubated with docetaxel for an additional 4 days. After treatment, the cells were washed with PBS, and 100 μL of medium was added back to the wells. To this, 10 μL of Tox8 was added to determine cell viability. The cells were incubated with the Tox8 reagent according to the manufacturer's instructions. The fluorescence was measured (λ_em _= 560 nm, λ_ex _= 590 nm) by a Spectramax Gemini Fluorescence Microplate Reader (Molecular Devices, Sunnyvale, CA). Wells without cells but with Tox8 were used as controls and subtracted from all treatments as background. Each experiment was performed in duplicate.

### Statistical analysis

Statistical analysis of the immunoblot data was performed using a non-parametric ANOVA (Kruskal Wallis) test. If significance was indicated, a Dunn's post test was used to determine significance between groups. Statistical analysis of siRNA-loaded nanogels plus docetaxel treated Hey or SK-OV-3 cells was compared to all controls (pNIPMAm, YSA-pNIPMAm, YSA peptide alone, and untreated cells). To determine significance between groups, a one-way ANOVA test was performed. If significance was indicated, a Tukey post test was performed to determine significance between sample groups. In all cases, significance was defined as P < 0.05.

## Results

### Down-regulation of EGFR in EphA2^+ ^ovarian cancer (Hey) cells by targeted siRNA-loaded nanogels

**Figure 1:**
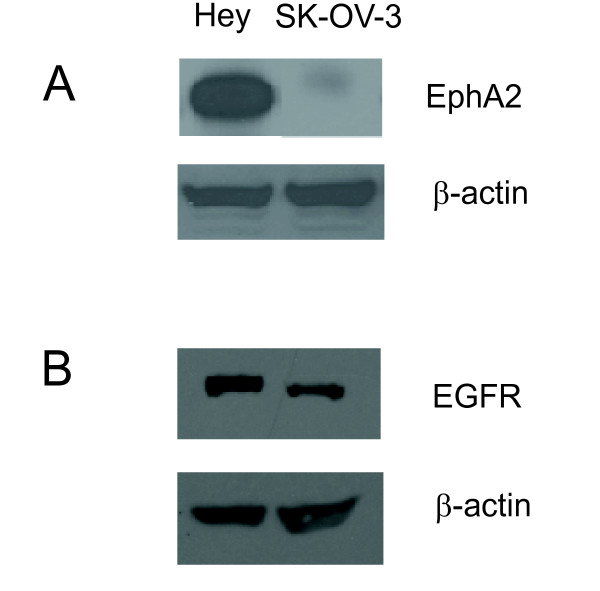
**Expression of EphA2 and EGFR in ovarian cancer cell lines**. **A**. Immunoblot analysis of EphA2 receptor expression in Hey and SK-OV-3 ovarian cancer cell lines. High expression of EphA2 is observed in Hey cells, but no expression is detected in the SK-OV-3 cell line. **B**. Expression of EGFR is observed in both the Hey and the SK-OV-3 cell lines as shown by immunoblotting. In both cases, β-actin expression was used to demonstrate equal loading of the protein samples so that the relative levels of each receptor could be compared between cell lines.

By coupling a peptide-mimetic (YSAYPDSVPMMS) of the EphA2 receptor's ephrin-A1 ligand to core/shell nanogels, we demonstrated previously the ability to target the delivery of siRNA to ovarian cancer (Hey) cells expressing the EphA2 receptor [26]. Importantly, these nanogels are nontoxic in both unmodified and targeted forms, and enabled the delivery of siRNA in serum-containing medium. To further establish the efficacy and specificity of this targeting method, we established a model using ovarian cancer cell lines either positive or negative for expression of EphA2 and positive for expression of EGFR. Figure 1A contrasts the high level of EphA2 receptor expression by Hey cells with the lack of EphA2 expression in the SK-OV-3 cancer cell line. Detection of EGFR was noted in both cell lines by immunoblotting (Figure 1B). Because of the observed differences in EphA2 expression levels, we hypothesized that the level of EGFR siRNA delivery and the subsequent decrease in EGFR expression in the cell lines would depend upon the presence of the EphA2 receptor as well as the concentration of siRNA loaded-nanogels added to the cells. Based upon this premise, reduction of EGFR expression in SK-OV-3 cells (EphA2 negative) should not be observed.

**Figure 2:**
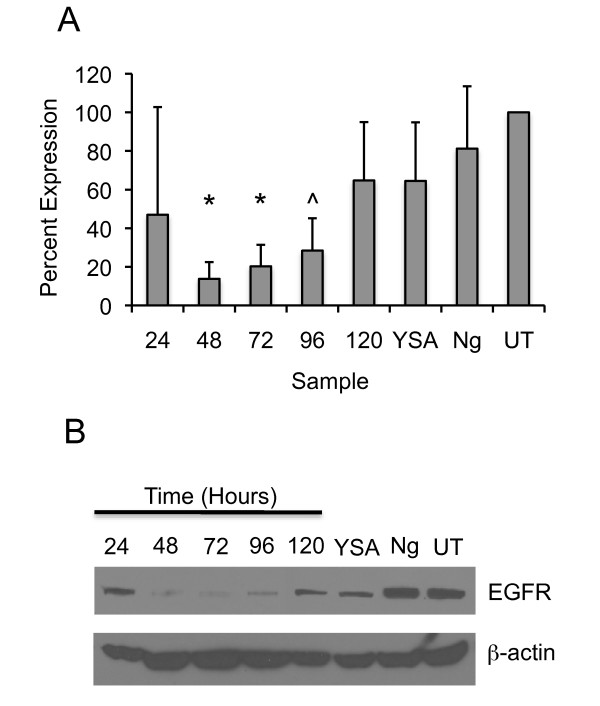
**Down-regulation of EGFR by siRNA-loaded nanogels**. **A**. Hey cells were examined for EGFR expression by immunoblotting of Hey cell lysates prepared from 24 to 120 hours after the addition of EGFR siRNA-loaded nanogels. Nanogels were loaded with siRNA at a concentration of 16.6 μg siRNA/mg of nanogels, and 1000 μg/mL of the loaded particles was added to 5 × 10^5 ^cells. Untreated (UT) cells were set at 100% expression of EGFR. Controls included unloaded/untargeted nanogels (Ng), and YSA peptide alone (YSA). Overall, treatment with EGFR siRNA significantly decreased receptor expression at 48 and 72 hours (*P < 0.01) and also at 96 hours (^P = 0.05). The error bars represent ± one standard deviation about the average value (n = 3). **B**. An immunoblot from one experiment out of three shows the decrease in EGFR expression over time. Controls are the same as those in (**A**).

**Figure 3:**
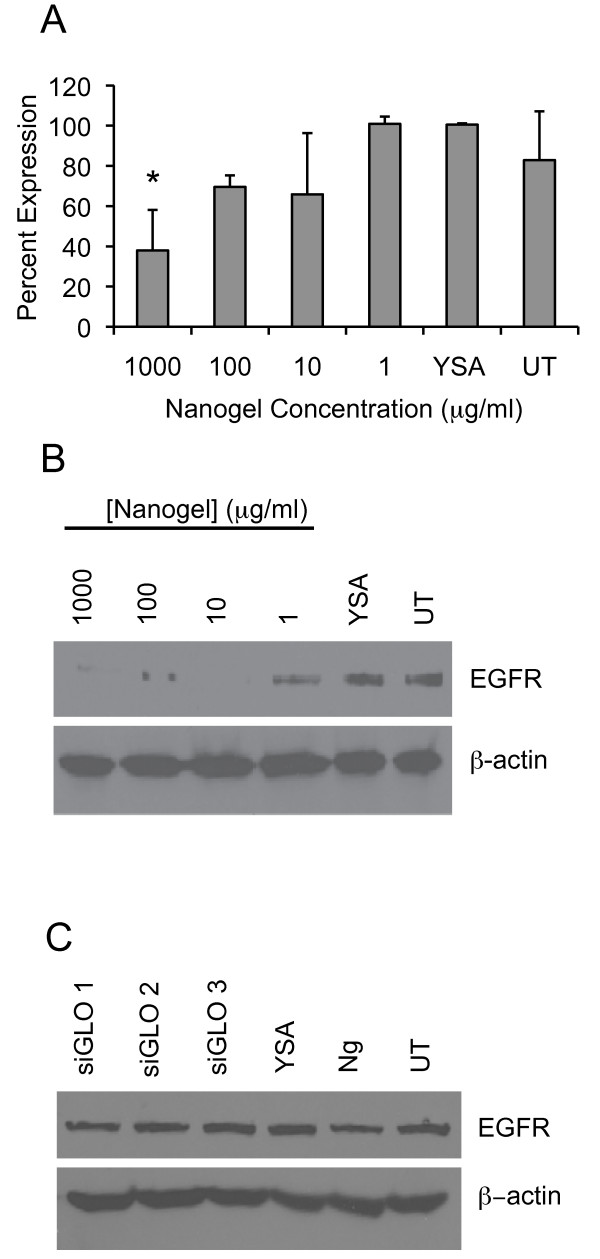
**Down-regulation of EGFR by different concentrations of siRNA-loaded nanogels**. **A**. A dose curve was established for EGFR expression by immunoblotting of Hey cell lysates. Nanogels were loaded with siRNA at a concentration of 16.6 μg siRNA/mg of nanogels, and 1000 μg/mL of the loaded particles was added to 5 × 10^5 ^cells. For other concentrations, nanogels were diluted serially by 10-fold (1000 μg/mL, 100 μg/mL, 10 μg/mL, and 1 μg/mL). All cells were harvested at 48 hours after the addition of the siRNA loaded nanogels. A significant decrease (*P < 0.01) in EGFR expression was observed at the highest nanogel concentration when compared to EGFR expression in untreated (UT) cells using the averaged values from three experiments. YSA peptide alone (YSA) was included as an additional control. **B**. An immunoblot from one of three experiments demonstrating EGFR expression in Hey cells after treatment with different concentrations of siRNA-loaded nanogels is shown. Note in the study shown that complete reduction in EGFR expression was observed when the concentration of nanogels used was as little as 10 μg/mL. **C**. Hey cells were treated with a non-specific siRNA, siGLO at a concentration of 1000 μg/mL of siGLO-loaded nanogels. Cells were harvested after 48 hours, and the levels of EGFR were examined by immunoblot. Differences in EGFR expression were not observed in siGLO-nanogel treated cells when compared with YSA peptide alone (YSA), nanogels (Ng), or untreated (UT) controls.

To test this hypothesis and measure the efficacy of the siRNA loaded nanogels in our system, we determined the time course of EGFR knockdown using EphA2 positive Hey cells. Lyophilized, YSA-targeted nanogels were loaded with EGFR siRNA by reswelling the particles in a concentrated solution of siRNA, as described [26]. This method results in high efficiency siRNA encapsulation (93 ± 1%) and approximately 70% retention of the siRNA after the first 12 hours. Long retention times may provide slow and continuous release of siRNA leading to prolonged reduction of the expressed target. Following siRNA encapsulation, the loaded nanogels were added to Hey cells and incubated at 37°C for four hours. In all experiments, we maintained a constant nanogel/cell ratio of 1 mg/mL of nanogels/5 × 10^5 ^cells, unless noted. Unincorporated nanogels were removed by washing and subsequent replacement of the cell culture medium. Treated cells were incubated for an additional 24, 48, 72, 96, and 120 hours to determine the time course of EGFR reduction by the nanogel-delivered siRNA. At each time point, the cells were lysed, and the samples were prepared for immunoblotting to determine the EGFR levels. Figure 2A shows the average (n = 3) percent decrease in EGFR expression at each time point. A significant decrease in EGFR expression (*P < 0.01) was observed at both 48 and 72 hours when compared to untreated (UT) controls. Significance (^P < 0.05) was also observed at the 96-hour time point when compared to untreated cells. These results indicate a maximum reduction of EGFR expression at 48 hours, and reexpression of EGFR beginning at approximately 72 hours. Expression gradually increased through 120 hours but did not return to pretreatment levels. This may be due to the slow but continuous release of siRNA from the nanogels. A slight decrease in EGFR expression was noted when the YSA peptide was used alone, which may be due to cross-talk between the EGFR and the EphA2 receptors [36]. Changes in EGFR levels may be due to loss of EphA2 as a result of YSA binding to the receptor and subsequent degradation of EphA2 (Dickerson, unpublished). Loss of EphA2 may disrupt EGFR expression through an as yet unknown mechanism. Note that this result was not observed in all studies performed (see Figure 3) and is under further investigation. In addition, a small decrease in EGFR expression was observed when cells were incubated with nanogels alone (Ng), but these decreases were not significant (P > 0.05). An immunoblot from one of three experiments is shown in Figure 2B. An immunoblot from a second experiment is presented in Additional file 1, Figure S1.

To determine the dose response for the delivery vector, EGFR siRNA-loaded nanogels were incubated with Hey cells using 10-fold serial dilutions of siRNA-loaded nanogels so that the nanogel concentration ranged from 1 μg/mL to 1000 μg/mL per 5 × 10^5 ^cells. Cells were harvested 48 hours after nanogel addition, and the cell lysates were analyzed by immunoblotting. In two out of three experiments, decreased levels of EGFR were observed at all concentrations. The average (n = 3) reduction in EGFR expression from all experiments is presented graphically in Figure 3A. A significant decrease (*P < 0.01) in EGFR expression was observed at the highest dose of delivered nanogels (1000 μg/mL) when compared to untreated controls (UT), and complete reduction of EGFR expression was observed with as little as 10 μg/mL of siRNA-loaded nanogels in some studies. An immunoblot demonstrating the reduction of EGFR with as little as 10 μg/mL of siRNA-loaded nanogels is shown in Figure 3B. The data shown is from one of three experiments. Data from a second experiment are presented in Additional file 2, Figure S2. We previously used a nonspecific siRNA, siGLO, to observe delivery of siRNA by YSA-targeted nanogels to Hey cells [26]. As a further control, treatment of Hey cells with YSA-targeted siGLO-loaded nanogels (siGLO) and analysis of EGFR expression levels did not result in loss of EGFR expression when treated cells were compared with the YSA peptide alone (YSA), untargeted nanogels (Ng) or untreated cells (UT), indicating the specificity of the EGFR siRNA (Figure 3C). The concentration of siGLO-loaded nanogels used was 1000 μg/mL.

**Figure 4:**
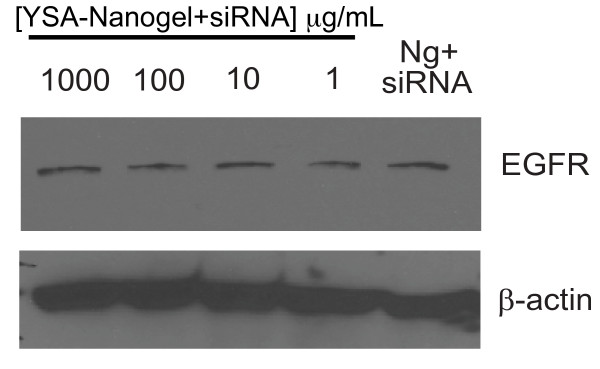
**Levels of EGFR in SK-OV-3 cells after treatment with YSA-targeted, siRNA-loaded nanogels**. EGFR expression in SK-OV-3 cells was determined by immunoblotting after the addition of several concentrations of YSA-targeted, siRNA-loaded nanogels (1 to 1000 μg/mL). All cells were harvested 48 hours after the addition of the siRNA-loaded nanogels. Untargeted but siRNA-loaded nanogels (Ng+siRNA) were included as a control. A change in EGFR expression was not observed between treatment groups as determined by immunoblotting.

The role of the peptide-targeted receptor, EphA2, in nanogel uptake, and the level of nonspecific nanogel incorporation into cells were explored through the use of an EphA2 negative cell line, SK-OV-3. Because these cells lack EphA2 expression, we hypothesized that the YSA-targeted nanogels would not be taken up by SK-OV-3 cells through receptor-mediated endocytosis of EphA2. Consequently, EGFR expression should not differ between targeted, siRNA-loaded nanogels and an untargeted, but siRNA-loaded control (Ng+siRNA). Any particle uptake could then be designated as nonspecific. For these studies, siRNA (1000 μg/mL of nanogels) was loaded into YSA-pNIPMAm nanogels and added to 5 × 10^5 ^SK-OV-3 cells. Ten-fold serial dilutions of the nanogels were carried out to assess the affects of nanogel concentration on the levels of EGFR. After 48 hours, harvested samples were examined for receptor expression by immunoblotting. As expected, expression of EGFR was not decreased after treatment with the loaded nanogels regardless of the concentration of nanogels used (Figure 4). Expression levels in SK-OV-3 cells treated with siRNA-loaded nanogels did not differ from an untargeted control, demonstrating the high specificity of the targeted nanogels for EphA2 positive cells but not for EphA2 negative cells.

### Epidermal growth factor receptor down-regulation in siRNA-loaded nanogel treated cells sensitizes ovarian cancer cells to docetaxel

**Figure 5:**
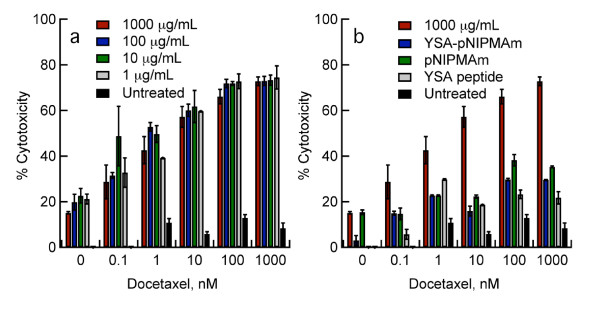
**Chemosensitization of Hey cells to docetaxel after exposure to YSA-targeted, siRNA loaded nanogels**. **A**. Hey cells were plated onto 96-well plates (1 × 10^4 ^cells/well) and allowed to adhere overnight at 37°C and 5% CO_2_. The medium was removed, and the cells were washed with PBS followed by replacement of the medium. Wells were set up in triplicate to include several concentrations of YSA-targeted, siRNA-loaded nanogels (1 to 1000 μg/mL). The cells were incubated with the nanogels for four hours. The cells were washed with PBS, the medium replaced, and the cells incubated for an additional 48 hours before addition of docetaxel in order to allow reduction of EGFR expression. Docetaxel was then added, and cells were incubated with the taxane for an additional 96 hours. The percent cytotoxicity (Tox 8 assay) was assessed compared to untreated cells. **B**. Chemosensitization of Hey cells treated with the highest dose of YSA-targeted, siRNA-loaded nanogels (1000 μg/mL) and increasing concentrations of docetaxel are compared to controls: Unloaded YSA-conjugated nanogels (YSA-pNIPMAm), unloaded pNIPMAm nanogels (pNIPMAm), YSA peptide alone, and untreated cells. Results represent the mean of two independent experiments; error bars represent standard deviation. All cells treated with siRNA-loaded, targeted nanogels were significantly different from controls at all doses of docetaxel except where noted [see Additional file 3, Tables S1-S4].

**Figure 6:**
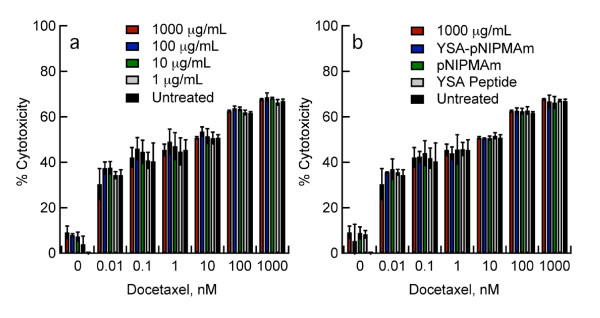
**Effects of increasing concentrations of docetaxel on SK-OV-3 cells treated with siRNA-loaded nanogels**. **A**. Effects of docetaxel on SK-OV-3 cells were tested alone or in combination with EGFR siRNA-loaded, YSA-targeted nanogels. The percent cytotoxicity (Tox 8 assay) was assessed at increasing concentrations of docetaxel and compared to untreated controls. **B**. Chemosensitization of SK-OV-3 cells treated with the highest dose of siRNA-loaded nanogels and increasing concentrations of docetaxel were compared to several controls. Controls included unloaded YSA-conjugated nanogels (YSA-pNIPMAm), unloaded pNIPMAm nanogels (pNIPMAm), YSA peptide alone, and untreated cells. Results are the mean of two independent experiments; error bars represent standard deviation. Chemosensitivity of nanogel treated SK-OV-3 cells did not differ significantly from all the controls examined (P > 0.05) at all doses of docetaxel.

Expression of EGFR is significantly related to chemosensitivity in many cancers. The concept of chemosensitization by EGFR blockade was provided by studies utilizing EGFR-blocking antibodies in combination with cisplatin or doxorubicin in human tumor xenografts [37,38]. Studies using a tyrosine kinase inhibitor against EGFR showed an increased sensitivity of ovarian cancer cell lines to paclitaxel after preincubation with the inhibitor [22]. To determine if our targeted delivery of EGFR siRNA to ovarian cancer cells could be used to increase cell line sensitivity to taxanes, Hey cells were incubated with EGFR siRNA-loaded nanogels for 48 hours to allow for maximum reduction in EGFR expression (see Figures 2A and 2B). After 48 hours, cells were treated with increasing concentrations of docetaxel (0.1 to 1000 nM), and the percent cytotoxicity was assessed. The results presented (Figure 5A) demonstrate the docetaxel sensitivity of treated Hey cells was almost 8-fold greater than untreated controls. While Hey cells treated with nanogel controls also showed increased chemosensitivity (Figure 5B), these changes were significantly less than those observed in cells treated with the YSA-targeted, siRNA-loaded nanogels (P < 0.01). Exceptions included the pNIPMAM and YSA-pNIPMAm controls where docetaxel concentrations were 0-0.1 (P > 0.05) at all nanogel concentrations examined, and for pNIPMAm and YSA-pNIPMAm controls when 1 μg/mL siRNA-loaded nanogels were delivered to cells followed by incubation with 1 nM docetaxel (P > 0.05) [see Additional file 3, Tables S1-S4]. Because SK-OV-3 cells lack expression of EphA2, and thereby lack the means for receptor-mediated endocytosis of the targeted nanogels, we did not expect the sensitivity of SK-OV-3 cells to docetaxel to be altered. Whereas an increase in cytotoxicity of the siRNA-loaded nanogel treated SK-OV-3 cells was noted as the concentration of docetaxel was increased, unlike the effect observed in the Hey cell line, sensitivity to the drug did not differ significantly from controls (P > 0.05) (Figures 6A and 6B). These results corroborate our earlier findings that EGFR levels are not decreased in this cell line after treatment with siRNA-loaded nanogels. It also substantiates the high specificity of our peptide-targeted system, and demonstrates little or no nonspecific uptake of nanogels by SK-OV-3 cells as shown by the constant levels of EGFR expression and unaltered chemosensitivity after nanogel treatment.

## Discussion

Novel therapies that interfere with specific molecular signaling pathways have potential as treatment options since they render cancer cells more sensitive to cytotoxic therapy. Although the role of EGFR in altering tumor chemosensitivity has not yet been fully elucidated, preclinical studies have suggested that blockade of EGFR, and the resulting reversal of chemoresistance in many tumor types is a viable strategy for treatment of cancers where frontline therapies have failed to induce a cure. Chemosensitization by EGFR inhibition was demonstrated in early studies using blocking antibodies in combination with cisplatin or doxorubicin in human tumor xenografts [37,38]. This same effect was later observed using small TKIs such as gefitinib (Iressa) [6,18,19,21]. Silencing of EGFR by RNAi is an alternative to anti-EGFR therapy, and this approach has already shown promising results [26-30]. However, further advances must be made to overcome problems with delivery and to insure specific delivery to the cells of interest.

While we previously demonstrated the specificity of YSA-targeted siRNA-loaded nanogels to cells expressing EphA2 [26], the studies presented here serve as further validation of EphA2 as a target for translatable therapeutic strategies. The EphA2 receptor is overexpressed in a variety of cancers including ~75% of ovarian malignancies, and expression of the receptor is associated with poor prognosis, increased metastasis, and decreased survival [39-41]. EphA2 shows limited expression in adults, with expression restricted to a few epithelial tissues [42]. Thus, due to its expression pattern, localization, and functional importance in treatment outcome, EphA2 is an attractive target for therapeutic agents in ovarian as well as other cancers [43]. Several approaches have been used to target EphA2 for cancer therapy either by taking advantage of the tumor-promoting function of EphA2 to modulate cell behavior and suppress tumor growth, or using EphA2 as a means to deliver agents, such as exogenous drugs, to tumor cells and the tumor microenvironment [44-47]. While these results demonstrate reduced tumor growth and limited metastatic spread, effectiveness of these treatments may depend upon tumor type and whether a particular tumor is dependent on EphA2-mediated pathways [48].

In this context, we noticed that treatment of Hey cells with the YSA peptide alone showed diminished EGFR expression when compared to untreated controls (Figures 2A and 2B) in some studies. Furthermore, Hey cells treated with the YSA peptide alone also showed an increased sensitivity to docetaxel when compared to untreated controls (Figure 5B). These differences were significant (P < 0.05) at docetaxel doses of 1 nM and higher. Interestingly, although we demonstrated silencing of EGFR with low doses of siRNA-loaded nanogels (10 μg/mL), the doses of docetaxel needed for chemosensitization in nanogel treated Hey cells were extremely low (≥1 nM). In addition, differences in chemosensitivity were not observed between low and high doses in our study even though presented results (Figure 3A and 3B) indicate that different levels of EGFR reduction were achieved depending upon the concentration of siRNA-loaded nanogels delivered. One possible explanation for this may be oncogene addiction where cancer cells are dependent upon or 'addicted' to one or several genes for maintenance of malignant phenotype and cell survival. Evidence for oncogene addiction to EGFR and members of the EGFR family has been described both at the cellular and clinical level [49]. The reader is referred to a review by Weinstein and Joe for a more in depth description behind the mechanisms of oncogene addiction [49].

Our results indicate that activation of EphA2 by the YSA peptide [33] and subsequent EphA2 degradation (Dickerson, unpublished) may lead to a reduction in EGFR expression indicating cross-talk between the two receptor signaling pathways. In fact, two recent studies have shown that EphA2 interacts with members of the EGFR receptor family, and these interactions may be important for targeted therapies involving EphA2 and EGFR [36,50]. Mice harboring ErbB2 (a member of the EGFR family) in mammary epithelium were sensitive to inhibition of EphA2 when compared to controls without ErbB2. EphA2 formed a complex with ErbB2 in both human and murine breast carcinoma cells, leading to enhanced signaling through Ras-MAPK activation and ultimately promoting tumor progression [50]. In addition, activated EGFR and the constitutively active EGFR type III deletion mutant (EGFRvIII) were shown to induce the expression of EphA2 in mammalian cell lines [36]. Loss of EphA2 expression reduced cell motility of EGFR-overexpressing cell lines. Miao et al [51] recently presented evidence that EphA2 serves as a common downstream effector molecule for growth factor signaling, including signaling through EGF and EGFR providing further evidence of an EGFR-EphA2 interaction. As a result, it is possible that loss of EphA2 may alter EGFR expression levels. Thus, the interaction of EphA2 with members of the EGFR family indicates a functional role for EphA2 in EGFR-expressing cancer cells. In our system, loss or reduction of EphA2 through interaction with YSA-functionalized nanogels may provide an enhanced effect over delivery of EGFR siRNA alone leading to a dual-targeting strategy for chemosensitization of ovarian tumors. While we did not observe reduction of EGFR in all experiments presented, the result is nonetheless intriguing and is under further investigation.

The ability of siRNAs to potently but reversibly silence genes *in vivo *has made them particularly well suited as a drug therapeutic. However, poor stability under physiological conditions limits the utility of systemic delivery of siRNA, and its high molecular weight (~13 kDa) and polyanionic nature prevent transport across the cell membrane, further compounding the problem of therapeutic application. Thus, delivery represents the main hurdle for broader development of siRNA therapeutics. To our knowledge, the work presented here along with our previous studies [26] provides the first description using targeted, poly(alkylacrylamide)-based nanogels for siRNA delivery. Furthermore, the core/shell nanogel delivery system employed here is readily amendable to selective surface functionalization by a variety of targeting molecules, offers a protective environment for sensitive cargo, and shows excellent targeting uptake and delivery in serum containing medium. The nanogel particles are also exceedingly simple to load, and extremely high (>90%) degrees of siRNA incorporation are observed. These properties and the low toxicity levels indicated thus far by this formulation, along with the low immunotoxcity demonstrated recently by Li et al [52], indicate the promise of overcoming some of the final obstacles hindering siRNA driven therapeutic strategies. Future studies investigating the *in vivo *delivery of siRNAs to tumors using nanogels, and the effect on chemosensitization will aid in the refinement of targeted siRNA delivery for treatment of ovarian cancer.

## Conclusion

The results presented herein demonstrate the therapeutic delivery of gene-specific siRNA cargo using peptide-functionalized nanogels, with the subsequent reduction of EGFR expression and increased chemosensitivity to docetaxel as a highly effective strategy for the sensitization of cancer cells to taxane chemotherapy. The broader significance of this work lies in the establishment of the YSA peptide and its EphA2 receptor target for the specific and efficient delivery of siRNA directly to cancer cells, and overcoming the main obstacle hindering therapeutic viability of siRNA treatment, that of delivery.

## Abbreviations

EGFR: epidermal growth factor receptor; EGF: epidermal growth factor; EphA2: erythropoietin-producing hepatocellular (Eph) receptor A2; RNAi: RNA interference; siRNA: small interfering RNA; YSA: 12 amino acid peptide (**YSA**YPDSVPMMS); TKI: tyrosine kinase inhibitor; pNIPMAm: poly(*N*-isopropylmethacrylamide).

## Competing interests

The authors declare that they have no competing interests.

## Authors' contributions

EBD, WHB, LAL, and JFM conceived the study. EBD, WHB, and MHS designed and carried out the studies. WHB and MHS synthesized and characterized the nanogels. LBK carried out experiments and assisted in data analysis. EBD was responsible for statistical analysis. EBD, LAL, and JFM were responsible for preparation of the manuscript. All authors read and approved the final manuscript.

## Pre-publication history

The pre-publication history for this paper can be accessed here:

http://www.biomedcentral.com/1471-2407/10/10/prepub

## Supplementary Material

Additional file 1 **Figure S1.** Down-regulation of EGFR by siRNA-loaded nanogels. An immunoblot from a separate experiment demonstrating reduction of EGFR expression over time is shown. Note that untargeted nanogels loaded with siRNA (Ng+siRNA) are used here as a further control. A decrease in EGFR expression is noted with this control indicating nonspecific uptake of the nanogels by the Hey cells. Click here for file

Additional file 2 **Figure S2.** Down-regulation of EGFR by different concentrations of siRNA-loaded nanogels. An immunoblot from a separate experiment demonstrating reduction of EGFR expression at the 1000 μg/mL dose of EGFR-siRNA loaded nanogels. Click here for file

Additional file 3 **Tables S1-S4**. Statistical analysis of siRNA-loaded nanogels + docetaxel treated Hey cells compared to all controls (pNIPMAm, YSA-pNIPMAm, YSA peptide alone, and untreated cells). To determine significance between groups, a one-way ANOVA test was performed. If significance was indicated, a Tukey post test was performed to determine significance between sample groups. Significance was defined as *P *< 0.05, and doses that were not significant are indicated as ns. Click here for file
