# Supplementary figures and images for: Chemosensitization of cancer cells by siRNA using targeted nanogel delivery
Source: BMC Cancer. 2010 Jan 11;10:10. doi: 10.1186/1471-2407-10-10 (PMC2820460; doi:10.1186/1471-2407-10-10)

Time (Hours)

24 48 72 96 120

YSA

Ng+siRNA

Ng

UT

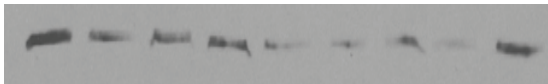

EGFR

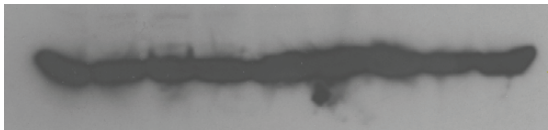

$\beta$ -actin

Supplement: Additional file 1 — Figure S1. Down-regulation of EGFR by siRNA-loaded nanogels. An immunoblot from a separate experiment demonstrating reduction of EGFR expression over time is shown. Note that untargeted nanogels loaded with siRNA (Ng+siRNA) are used here as a further control. A decrease in EGFR expression is noted with this control indicating nonspecific uptake of the nanogels by the Hey cells. [file 1471-2407-10-10-S1.PDF]

[Nanogel]  $\mu\text{g/ml}$

---

1000

100

10

1

YSA

UT

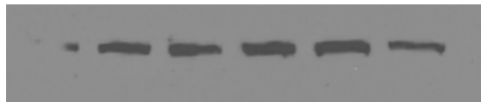

EGFR

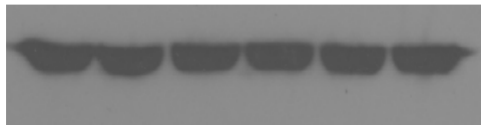

$\beta$ -actin

Supplement: Additional file 2 — Figure S2. Down-regulation of EGFR by different concentrations of siRNA-loaded nanogels. An immunoblot from a separate experiment demonstrating reduction of EGFR expression at the 1000 μg/mL dose of EGFR-siRNA loaded nanogels. [file 1471-2407-10-10-S2.PDF]
