# Supplementary material for: Chemosensitization of cancer cells by siRNA using targeted nanogel delivery
Source: BMC Cancer. 2010 Jan 11;10:10. doi: 10.1186/1471-2407-10-10 (PMC2820460; doi:10.1186/1471-2407-10-10)
Supplement: Additional file 3 — Tables S1-S4. Statistical analysis of siRNA-loaded nanogels + docetaxel treated Hey cells compared to all controls (pNIPMAm, YSA-pNIPMAm, YSA peptide alone, and untreated cells). To determine significance between groups, a one-way ANOVA test was performed. If significance was indicated, a Tukey post test was performed to determine significance between sample groups. Significance was defined as P < 0.05, and doses that were not significant are indicated as ns. [file 1471-2407-10-10-S3.PDF]

1000 µg/mL Nanogels+ siRNA (Supplementary Table 1)

**Docetaxel [nM]**	**Untreated**	**pNIPMAm**	**YSA-pNIPMAm**	**YSA**
0	<0.001	>0.05 ns	>0.05 ns	<0.01
0.1	<0.001	>0.05 ns	>0.05 ns	<0.001
1	<0.001	<0.001	<0.05	<0.01
10	<0.001	<0.001	<0.001	<0.001
100	<0.001	<0.001	<0.001	<0.001
1000	<0.001	<0.001	<0.001	<0.001

100 µg/mL Nanogels+ siRNA (Supplementary Table 2)

**Docetaxel [nM]**	**Untreated**	**pNIPMAm**	**YSA-pNIPMAm**	**YSA**
0	<0.001	>0.05 ns	>0.05 ns	<0.01
0.1	<0.001	>0.05 ns	>0.05 ns	<0.001
1	<0.001	<0.001	<0.01	<0.001
10	<0.001	<0.001	<0.001	<0.001
100	<0.001	<0.001	<0.001	<0.001
1000	<0.001	<0.001	<0.001	<0.001

10 μg/mL Nanogels+ siRNA (Supplementary Table 3)

**Docetaxel [nM]**	**Untreated**	**pNIPMAm**	**YSA-pNIPMAm**	**YSA**
0	<0.001	>0.05	>0.05	<0.01
0.1	<0.001	>0.05	>0.05	<0.001
1	<0.001	<0.001	<0.01	<0.001
10	<0.001	<0.001	<0.001	<0.001
100	<0.001	<0.001	<0.001	<0.001
1000	<0.001	<0.001	<0.001	<0.001

1 μg/mL Nanogels+ siRNA (Supplementary Table 4)

**Docetaxel [nM]**	**Untreated**	**pNIPMAm**	**YSApNIPMAm**	**YSA**
0	<0.001	>0.05	>0.05	<0.01
0.1	<0.001	>0.05	>0.05	<0.001
1	<0.001	>0.05	>0.05	<0.01
10	<0.001	<0.001	<0.001	<0.001
100	<0.001	<0.001	<0.001	<0.001
1000	<0.001	<0.001	<0.001	<0.001
